# A Cadaveric Case Study on the Abdomen: A Temple of Surprises

**DOI:** 10.7759/cureus.71618

**Published:** 2024-10-16

**Authors:** Kristi Fung, Mathangi Rajaram-Gilkes, Taylor Moglia, Finn G Rieker, Catherine Falkenstein

**Affiliations:** 1 Medicine, Geisinger Commonwealth School of Medicine, Scranton, USA; 2 Anatomy, Geisinger Commonwealth School of Medicine, Scranton, USA

**Keywords:** aaa with cabg, abdominal aortic aneurysm, endovascular aortic repair (evar), evar with femoro-femoral bypass graft, femoro-femoral bypass graft, umbilical hernioplasty

## Abstract

Femoro-femoral bypass grafts (FFBG) are performed to connect the major vessels of the lower extremities, such as the femoral arteries, to treat patients who have injured or occluded iliac arteries. Typically, patients with multiple comorbidities, such as heart failure, aneurysms, or diabetes, have a significantly higher risk of complications for open, invasive procedures to correct lower limb ischemia. This graft poses as an effective, less invasive option to treat lower-limb ischemia for higher-risk patients. This case study presents a finding of FFBG in an 82-year-old male cadaver during cadaveric dissection in the gross anatomy lab at Geisinger Commonwealth School of Medicine in Scranton, Pennsylvania. Based on the initial findings of cardiomegaly with a triple coronary artery bypass graft (CABG) and pulmonary hypertension in the thoracic cavity and evidence of massive umbilical hernioplasty involving extensive mesh repair, our initial assumption of an FFBG placement in this cadaver was to increase perfusion to lower limbs, circumventing the need for surgical intervention due to the above-mentioned comorbidities, which act as risk factors. However, the discovery of a massive abdominal aortic aneurysm (AAA) measuring 26 cm in circumference with evidence of dissection of its wall and the presence of a stent within the aorta and common iliac arteries placed there as an endovascular aneurysm repair (EVAR) procedure came as a surprise. Publication of such findings provides awareness to curious individuals about the existence of multiple health concerns an individual suffers and how the medical as well as surgical teams work together to provide optimal treatment care to improve their standard of living and prolong their lifespan.

## Introduction

Femoro-femoral bypass graft (FFBG) is a surgical procedure that is predominantly done in the case of an injured or occluded iliac artery [1]. This procedure involves the placement of a vascular prosthesis that connects the major vessels of the lower extremities, such as femoral arteries, mainly to treat patients with severe claudication or ischemia [2]. Patients with large abdominal aortic aneurysms (AAA) also undergo this bypass procedure to improve circulation in the lower extremities [3]. During the first year of medical education, the discipline of gross anatomy involved dissecting an 82-year-old male cadaver. The regions dissected were in the order of back, and spinal cord with laminectomy, upper limbs, lower limbs, thorax, head and neck regions, abdomen, and pelvic regions. In this sequence of regional dissection, certain procedures done on this cadaver were observed and posed a challenge as to the sequence of events that might have occurred and warranted the various surgical procedures. As the abdomen and pelvis were opened towards the end, the identification of the surgical procedures performed on this person created great curiosity and challenge to interpret the interaction of the medical and surgical fields to extend the lifetime of this person and provide him with a better quality of life. Here we provide the findings, in the order of discovery, coronary artery bypass grafts (CABG), a large umbilical hernioplasty, a FFBG, and a AAA with endovascular aneurysm repair (EVAR), during our dissection as we progressed through the regions described above. These findings, along with the clinical interpretation, were quite eye-opening in relation to human anatomy affected by disease processes and the surgical measures that were taken to address the relevant complaints to increase the life expectancy and quality of life of patients.

## Case presentation

During the process of dissection during the first year of medical education in the gross anatomy lab of Geisinger Commonwealth School of Medicine in Scranton, Pennsylvania, the following observations were made in an 82-year-old male cadaver. The dissections covered regions in the order of back, and spinal cord with laminectomy, upper and lower limbs, thorax, head, neck, abdomen, and pelvis. Dissection of the thoracic cage was uneventful until the scar over the sternum with metallic wire closures was observed, indicating open heart surgery. Further exploration revealed a widened mediastinum within the thoracic cavity cradling an enlarged heart with evidence of CABGs. Further observation of scars in the anterior abdominal wall and femoral regions bilaterally led to extensive and careful dissection, resulting in several findings that are described in the following sections. The dissections were performed by a group of four students supervised by course faculty. The dissection took place over a period of weeks as various regions were covered in different blocks, and the final regions of the posterior abdominal wall and pelvis culminated in an endeavor to disseminate our findings.

Thoracic cage and cavity

On the exterior of the thoracic cage, steel wires were found intermittently bringing the sternum together, indicating that an open heart surgery was performed. Dissection was done to remove the anterior aspect of the second to seventh ribs bilaterally to expose the thoracic cavity. Exploration of the middle mediastinum revealed an enlarged heart with evidence of CABG procedures performed in the past. Removal of the heart showed severe cardiomegaly with enlarged and dilated chambers and thinned-out ventricular walls. Both lungs showed hyperemic changes, perhaps indicating severe congestive changes during the time of death. The arch of the aorta and the descending thoracic aorta appeared uniformly widened, but not to a significant extent, as it entered through the diaphragm into the abdominal cavity. Due to contributions to other research procedures, the specimens of the heart and lungs were moved to another campus and were unavailable for pictures to present in this paper.

Abdomen

Anterior Abdominal Wall

**Figure 1:**
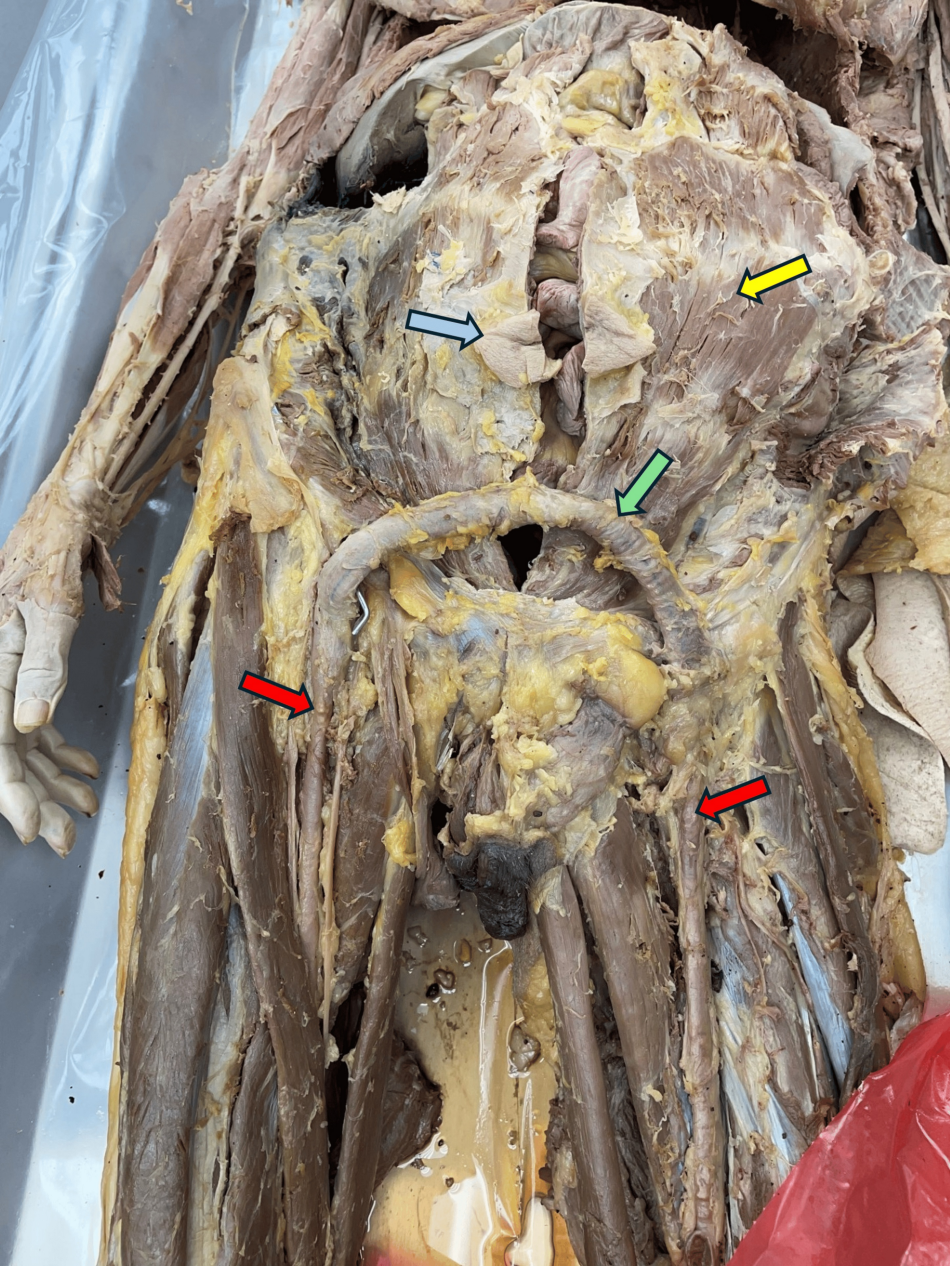
Image of the abdomen and the upper thighs after the removal of the skin The yellow arrow indicates the rectus abdominis muscle, the blue arrow indicates the umbilicus, the green arrow indicates the femoro-femoral bypass graft, and the red arrow indicates the femoral arteries.

The external appearance indicated surgical wounds to the anterior abdominal wall close to the umbilicus in a paramedian fashion on the left side. The image below shows the abdominal wall muscles and the FFBG extending between the femoral arteries bilaterally. The rectus abdominis muscles were thinned out and widened, indicating chronic stretching over the abdominal wall (Figure 1).

Suprapubic Region

**Figure 2:**
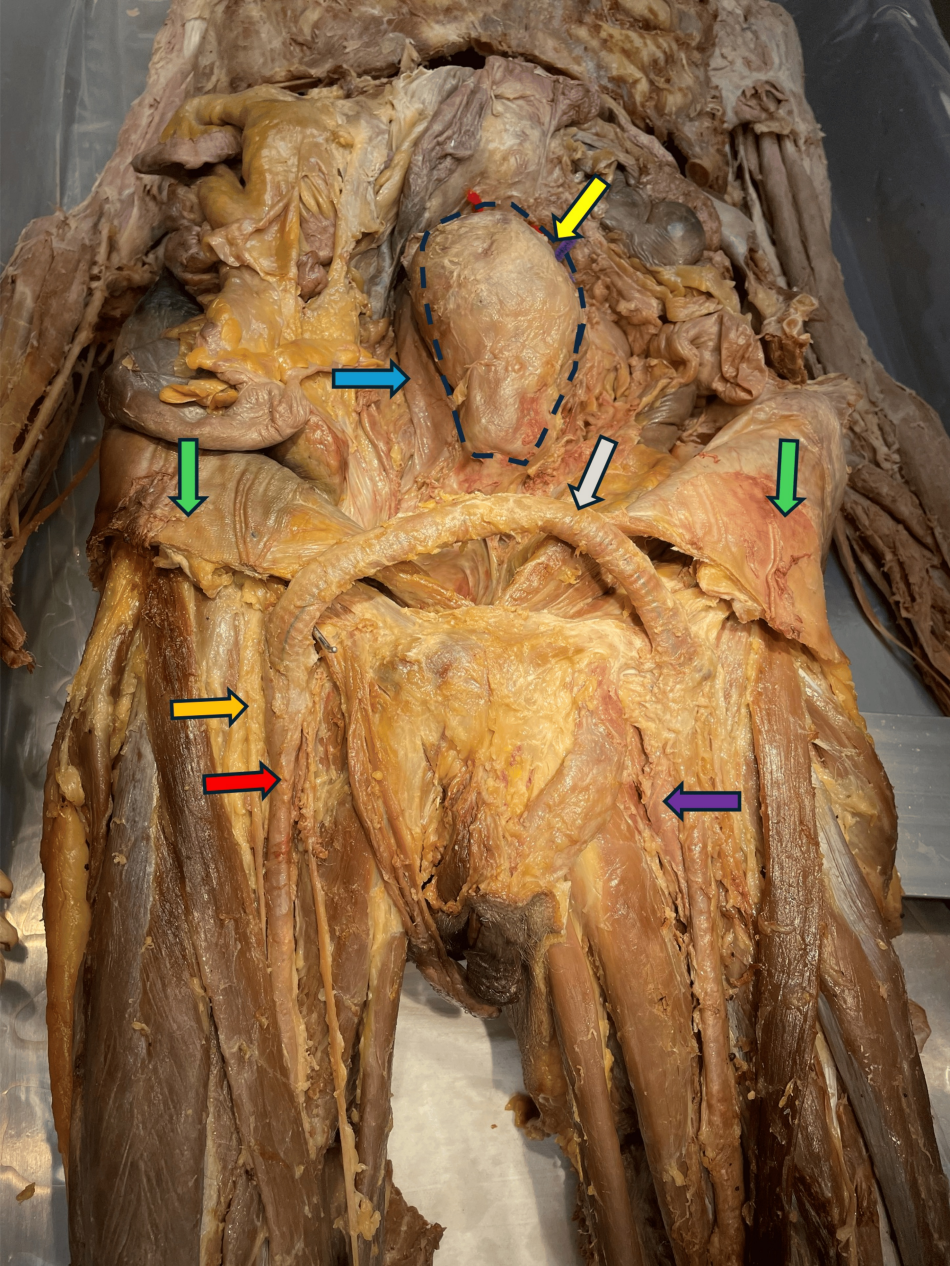
Image showing an aortic aneurysm and hernioplasty on the anterior abdominal wall The yellow arrow indicates the aneurysm (outlined in blue dashes), the blue arrow indicates the inferior vena cava, the green arrow indicates hernioplasty sites, the orange arrow indicates the femoral nerve, the red arrow indicates the femoral artery, the purple arrow indicates the femoral vein, and the gray arrow indicates the femoro-femoral bypass graft.

The parietal layer of the peritoneum revealed hernioplasty around the umbilical region, which normally indicates the repair that involves reinforcement of the herniorrhaphy with a large mesh covering a wide area on the inner aspect of the anterior abdominal wall (Figure 2). In the suprapubic region, there were small incision wounds bilaterally over the femoral triangle regions. Subcutaneously, a tubular graft was observed connecting the femoral arteries bilaterally, measuring 13 inches. Exploration of the femoral arteries showed that they only connected the two femoral arteries. Granulation tissue was observed to seal the wounds seamlessly, indicating that the procedure was not done recently. There was also adipose tissue adherent to the graft throughout its length. The aneurysm has been outlined by a blue dotted line. The inferior vena cava can be seen running along the aorta to its right. No anomaly was associated with the inferior vena cava. It received the drainage of the gonadal vein on the right normally (Figure 2).

Posterior abdominal wall

Aneurysm

**Figure 3:**
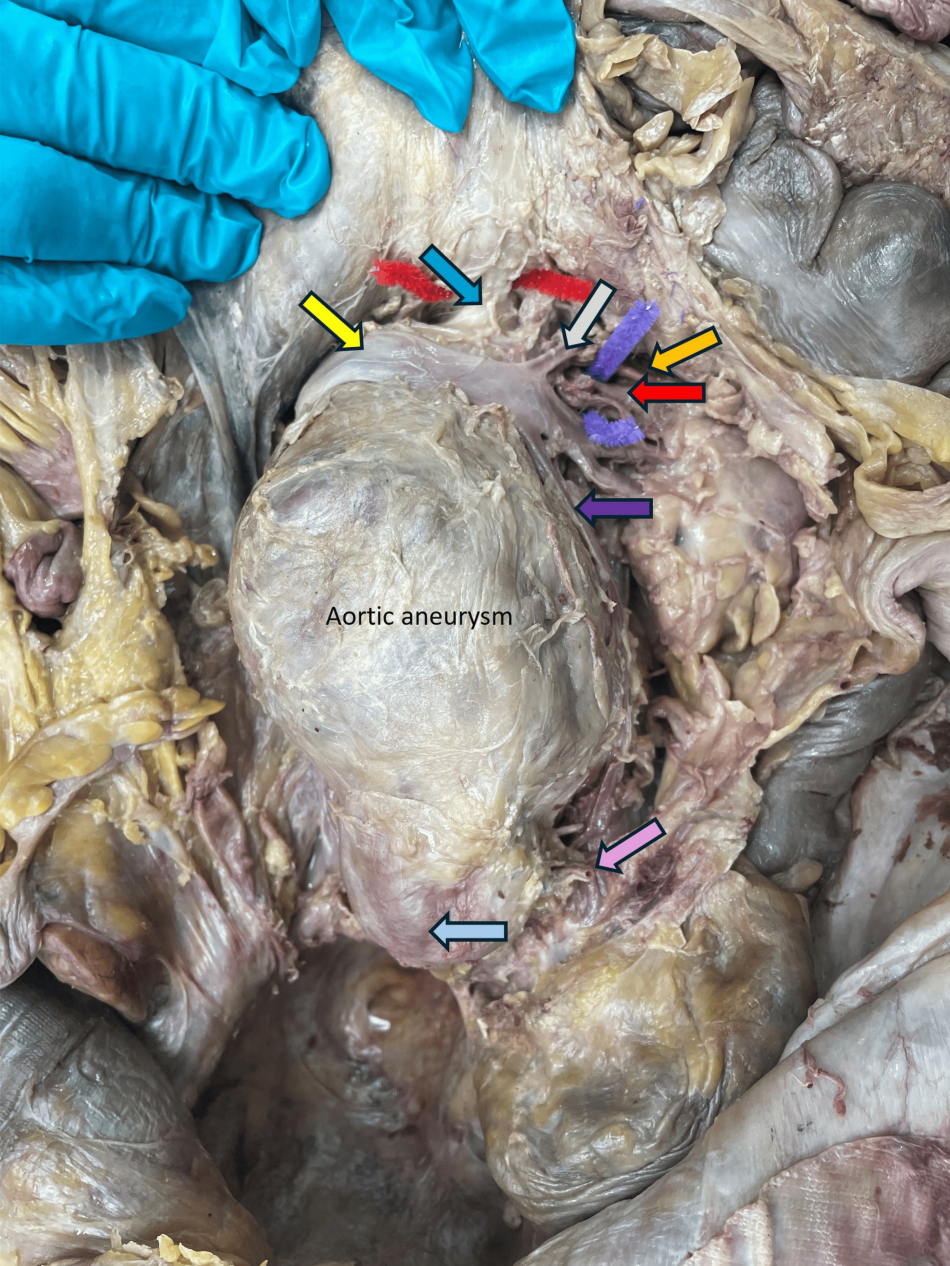
Image showing the aortic aneurysm and the blood vessels adjacent to it The yellow arrow indicates the left renal vein, the dark blue arrow indicates the superior mesenteric artery, the gray arrow indicates the left suprarenal vein, the orange arrow indicates the left suprarenal gland, the red arrow indicates the left renal artery, the purple arrow indicates the left gonadal vein, the pink arrow indicates the inferior mesenteric artery, and the pale blue arrow indicates the lower end of the aorta.

On the posterior abdominal wall, a large pear-shaped bulge of the abdominal aorta was observed to be extending from beneath the left renal vein to an inch above its termination. The aneurysm extended to a length of 16.5 cm and ended 1 cm above the termination of the aorta. As to the vertebral levels, it extended from L2 to the upper border of the L4 vertebral level. Filled with a thrombus and firm to touch, the aneurysm appeared to be pear-shaped. At its maximum width, the circumference was 26 cm and the diameter was 8 cm. The left renal vein was observed to be passing between the superior mesenteric artery (SMA) and the aorta. The origin and caliber of SMA were normal. The left renal artery was short, and segmental branches were seen to enter the hilum of the kidney. A midline incision was made on the aneurysm from beneath the left renal vein as it crossed over the aorta, and the cut was extended to its termination (Figure 3).

**Figure 4:**
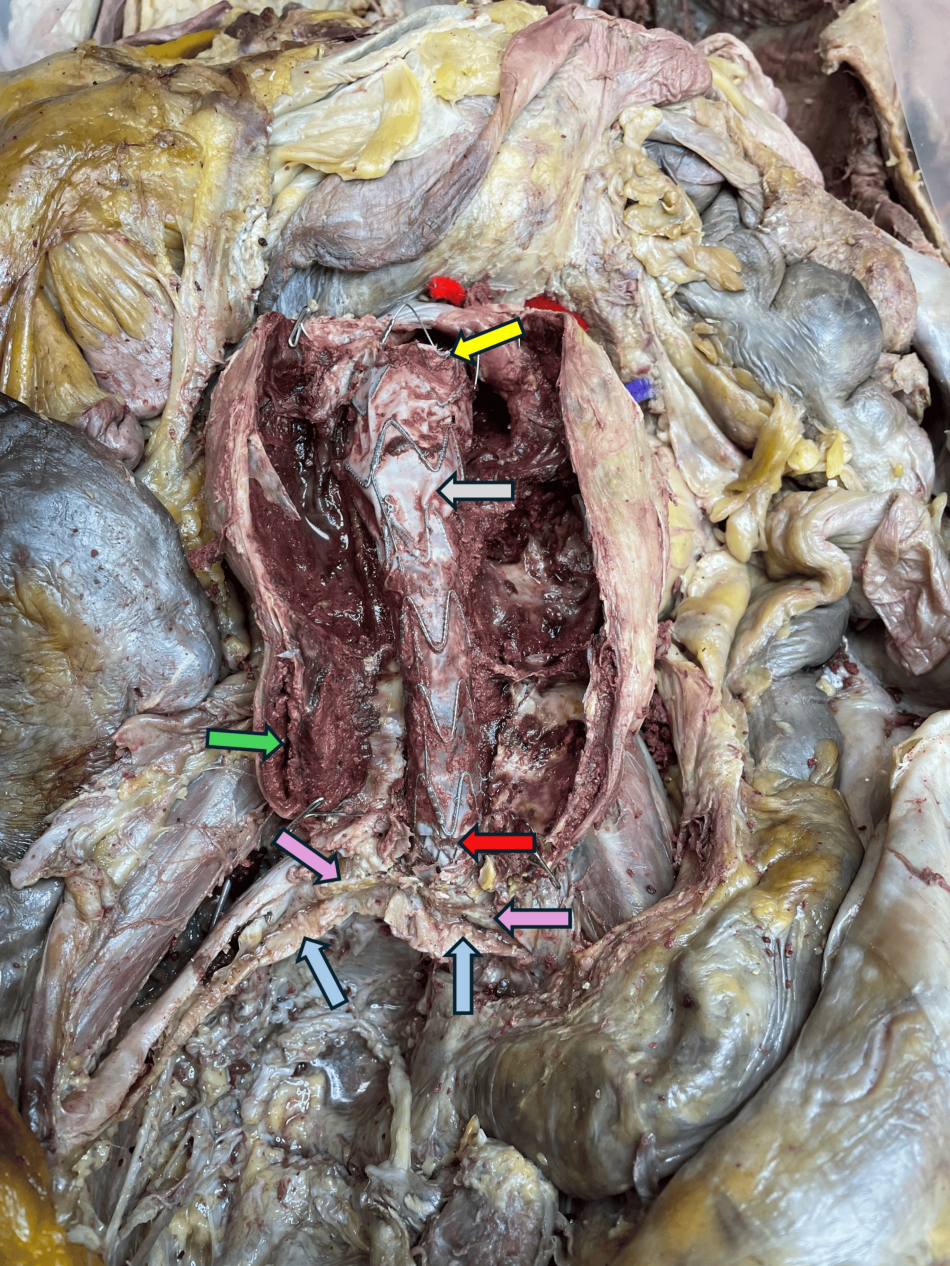
Image showing the endovascular stent graft within the abdominal aorta and proximal common iliac arteries The yellow arrow indicates the upper limit of the stent in the aorta, the gray arrow indicates the stent graft placement, the red arrow indicates the lower limit of the stent in the aorta, the blue arrow indicates the right and left common iliac arteries (cut open), the green arrow indicates the dissection observed in the wall of the aorta, and the pink arrow indicates the stents in the common iliac arteries.

The thrombus was removed to reveal EVAR done on this person. The graft began from just beneath the left renal vein level and extended into the proximal one-third of both common iliac arteries. The wall of the aneurysm towards the lower end revealed a slit between a thickened tunica intima and tunica media for an extent of two and a half inches. The wall of the remainder of the dilated aneurysm segment did not reveal dissection (Figure 4).

Posterior abdominal wall

Blood Vessels and Other Structures

Superficial observation of the posterior abdominal wall showed no disturbance to the peritoneal lining, scarring, or adhesions. The branches of the aorta and other blood vessels close to it were examined. At the upper limit of the aneurysm, the left renal vein was observed to cross over the aorta to drain into the inferior vena cava. Close to the left kidney, it received the suprarenal and gonadal veins. The renal artery was short and arose from the aorta, and the segmental branches were seen entering the hilum of the left kidney. The left gonadal artery was observed to arise from the left renal artery instead of the abdominal aorta. The inferior mesenteric artery rose one inch above the termination of the aorta. However, the branch was small in diameter and was pushed to the left side due to the aneurysm (Figure 3). Lumbar arteries were identified bilaterally and were also found to be of smaller caliber than normal. The pattern of venous return, however, remained normal and the vessels were of normal caliber. The sympathetic chain on the left seems to run adherent to the adventitia of the aorta throughout the length of the aneurysm. The right sympathetic trunk was observed to be normal. The celiac trunk and the branches were found to be normal. The liver, spleen, and pancreas appeared healthy and normal. Portal vein formation was observed to be normal.

Pelvic cavity

The pelvic cavity appeared normal with no surgical inventions to the naked eye. The peritoneal reflections were normal. The ureters were normal and coursed bilaterally to enter the pelvic cavity, crossing the bifurcation of common iliac arteries to run towards the bladder. However, the common iliac arteries were hard on palpation, and the presence of a bypass graft within the abdominal aorta led to the dissection of the common iliac arteries to find the extension of the same into the proximal segments of both common iliac arteries (Figure 4). The caliber of pelvic vessels was narrower than what one would normally expect. Prostate, seminal vesicles, and ductus deferens appeared normal.

## Discussion

The ascending aorta originates directly from the left ventricle at the aortic valve, and immediately the coronary arteries branch off before the aorta ascends to the T4 vertebral level, curving posteriorly leftwards, forming the aortic arch, at which point the brachiocephalic, left common carotid, and left subclavian arteries branch off [4]. As the aortic arch descends, it transitions into the thoracic aorta, which gives rise to the visceral pericardial, bronchial, esophageal, and mediastinal branches, as well as the parietal intercostal, subcostal, and superior phrenic branches [4]. The thoracic aorta traverses the diaphragm via the aortic hiatus at the T12 vertebral level, becoming the abdominal aorta, which gives rise to, in descending order, the celiac trunk, superior mesenteric artery, left and right renal arteries, and inferior mesenteric artery [4]. The abdominal aorta bifurcates into the left and right common iliac arteries at the L4 vertebral level [4], which each bifurcate into internal and external iliac arteries anterior to the sacroiliac joint, the latter of which exits the pelvis deep to the inguinal ligament, becoming the common femoral artery [5]. The common femoral artery branches into the superficial and deep femoral arteries; the deep femoral artery gives rise to the medial and lateral circumflex arteries and ultimately terminates as the perforating branches. These arteries supply the lower extremities, with the superficial femoral artery providing most of the blood flow to the lower legs and the deep femoral artery and its branches supplying the hip and the femur regions [6]. Occlusion of either or both of the iliac arteries and their branches most often presents as intermittent claudication of the lower extremities, especially with exertion, with higher-grade stenosis causing more proximal pain [6,7,8]. Limb-threatening obstruction is associated with pain at rest, non-healing ulceration, and gangrene [6,7,8]. Bilateral obstruction at the level of the aortic bifurcation into the iliac arteries presents with the classic Leriche syndrome triad of buttock claudication, erectile dysfunction, and decreased femoral pulses [7].

Abdominal aortic aneurysms are dilations of large vessels that exceed 3.0 cm, or 50% of the normal diameter [9,10]; AAAs are more common in men, and the incidence increases with age, most commonly being diagnosed in patients over the age of 65, which is consistent with the cadaver presented here [11]. Risk factors for acquiring an AAA include cigarette smoking, obesity, male gender, older age, family history, and cardiovascular disease [12]. Rupture of an AAA is associated with exceptionally high mortality, causing death in about 90% of patients, and was the cause of about 3% of deaths in the United States in 2017 [10,13]. Larger AAAs infer an increased risk of rupture, with surgical repair indicated for all ruptured or symptomatic aneurysms, rapidly expanding AAAs, asymptomatic aneurysms exceeding 4 cm or twice the diameter of the normal aorta, or declining quality of life [14]. Survival rates increase to about 50% with surgical intervention, though many patients die from rupture before they are aware of their condition or can undergo surgical repair [12]. People living with an AAA are at an increased risk of cardiovascular events, including chronic heart failure [15]. Several studies have associated AAAs with increased left ventricular wall thickness, reduced left ventricular systolic function, higher filling pressures on the left ventricle, ischemic heart disease, increased arterial stiffness, and hypertension, connecting AAA with general vascular disease [16,17]. This cadaver’s symptoms of congestive heart disease, coronary and femoral artery occlusions, and cardiomegaly are consistent with evidence of systemic vascular disease that has been highly associated with AAA in elderly male patients. Given that the cadaver’s AAA was 8 cm, greatly exceeding the threshold for surgical intervention, this person would have likely been recommended to receive surgical repair if the AAA had been identified.

For patients with a history of cardiovascular disease such as congestive cardiac failure, non-cardiac procedures carry an increased risk of mortality from adverse cardiac events, which may make them unfit for surgery. Patients with a history of unstable angina, decompensated heart failure, hemodynamically important valvular heart disease, or a history of myocardial infarction within 60 days have an increased risk of cardiac complications; these conditions predispose the patient to myocardial ischemia due to volume shifts, blood loss, increased perioperative myocardial oxygen demand due to surgical stress demand, and platelet activation during surgery [18]. Lerman et al. found that amongst patients with heart failure, there was a 5.7% risk of 30-day postoperative complications in comparison to a 2.7% risk among patients without heart failure [19]. Within one year, one in five high-risk patients undergoing major non-cardiac surgery will develop one or more heart complications, as surgery can trigger cardiac events such as myocardial infarctions, heart failure, and heart rhythm disturbances, especially in elderly patients with significant comorbidities who undergo surgery [20]. Complications to surgery may go unaddressed due to a lack of symptoms; for instance, patients are often given pain medication following surgery and fail to express classic signs of myocardial infarctions that may alert physicians to the underlying cardiac event. 

Furthermore, anesthesia use in patients with significant histories of heart disease during surgery may predispose them to adverse cardiovascular events. In healthy patients, anesthesia causes cardiac depression and hemodynamic instability, which is compounded in patients with congestive heart failure, as they are unable to perform the typically compensatory mechanisms that maintain a normal cardiac output and heart rate [21]. When patients are given an increased dosage of inhaled anesthetics, cardiac output is reduced; healthy individuals can respond with an increased heart rate. Those who are elderly, have comorbidities, and/or are under concurrent medications may inhibit this compensatory mechanism, resulting in a significant decrease in cardiac output and more pronounced cardio-depressant effects [21]. Inhaled anesthetics may depress contractility and cause a decreased heart rate, which places further strain on the patient’s cardiac function [22]. The elderly, especially those with cardiac diseases, often suffer from hemodynamic instability due to vascular and myocardial stiffening, sympathetic overactivity, comorbidities, and concurrent medication use. Consequently, this places them at higher risk of experiencing significant cardiovascular events following anesthesia use during surgery. 

Patients with heart failure are also at a higher risk for the development of thromboembolic events in comparison to the general population, due to abnormal blood flow, poor cardiac contractility, low cardiac output, and an increased hypercoagulable state due to increased platelet activation [23,24]. Surgery and its associated prolonged immobilization during recovery place patients with a history of congestive heart failure at a greater risk for the development of superficial blood clots or deep-vein thrombosis. Patients are often given antithrombotics and anticoagulants to combat and prevent the development of such conditions, which may result in an increased bleeding risk both during and after the surgical procedure [25]. 

The femoro-femoral crossover bypass (FCB) technique serves as a treatment option for patients with unilateral occlusive iliac artery disease [26]. Although it is typically performed for high-risk cases, it can be indicated for low-risk patients as an alternative to more invasive reconstructive surgeries [27]. The five-year patency rates are estimated to be as high as 60% [28] and even up to 70% for primary patency rates and 85% for secondary patency rates [29]. Another 10-year study found that after five years, primary and secondary patency rates were 54% and 84%, respectively [29]. In addition, this less invasive technique has been shown to improve the hemodynamic status of high-risk patients [28]. Hemodynamic instability refers to a mismatch between the oxygen demand and oxygen delivery to peripheral tissues. Patients who are considered hemodynamically “unstable” often have impaired blood circulating volume, cardiac function, and/or vascular tone in peripheral tissues, which can contribute to a variety of pathologies such as organ failure and death [30]. Typically, patients with lower limb ischemia have associated comorbidities such as hypertension, dyslipidemia, diabetes mellitus, chronic kidney disease, and atherosclerotic disease and are thus at an increased risk for thromboembolic events, which can further impair blood circulation [31]. Surgery greatly increases the risk for thromboembolic events, such as a deep vein thrombosis or a pulmonary embolism [32], hence less invasive techniques should be utilized for patients already at high risk for these complications. As shown in this high-risk cadaver with multiple comorbidities, a FFBG serves as a great alternative to open surgical interventions that may harm the patient more than it will benefit them. 

There are two surgeries available for the treatment of abdominal aortic aneurysms. Of the two, open surgical repair (OSR) is more common; the procedure entails making a cut to the location of the aneurysm, removing the aneurysm, and sewing a graft into its place. It is a more invasive procedure and involves a longer recovery time [33]. The alternative procedure is EVAR, which is performed by cardiac catheterization. A small cut is made in the groin where a stent graft is placed and sent to the abdominal aorta. The stent expands and attaches to the aortic walls to form a seal between the stent graft and the vessel wall to prevent blood from entering. For patients who meet specific anatomic criteria, EVAR is the preferred procedure, as it results in decreased blood loss, does not require cross-clamping of the aorta, and has a shorter recovery time. The main disadvantage of EVAR involves complications that may require further surgical intervention, and most patients require lifelong surveillance with CT scans following the procedure [34].

The EVAR procedure has a lower mortality rate than OSR, but the OSR mortality rates have decreased in the last 10 years [35]. Both procedures carry similar risks and complications, which include but are not limited to hemorrhages due to excessive blood loss, myocardial infarctions, strokes, and thromboembolic events. Factors associated with a worse prognosis include advanced age, more severe disease, the need for emergent intervention due to a ruptured aneurysm, and the presence of comorbidities such as cardiovascular disease, chronic obstructive pulmonary disease, and diabetes mellitus [35]. Open surgical repair has a higher risk of damage to other organs due to reduced blood flow during surgery. Ischemia is thought to occur at higher rates in OSR because of endograft limb occlusions, which often manifest as rest pain or a continuous burning pain in the lower extremities that occurs during elevation, intermittent claudication, or decreased femoral pulses [35]. It was also found that OSR sometimes manifests itself in buttock claudication due to the intraoperative ligation of the internal iliac artery, resulting in reduced blood flow [36]. Complications associated with the EVAR procedure include the danger of endoleaks, improper stent placement, stent migration, and graft infection [34]. Endoleaks occur when the graft fails to prevent blood from entering the aneurysmal sac; the necessity of surgical intervention to repair the endoleak depends on the type of endoleak [37].

It is our opinion that for elderly patients with a significant history of cardiovascular disease, such as congestive heart failure, signs of coronary artery disease, and congestive changes to the lungs, undergoing the OSR or EVAR procedure would not be ideal due to the great likelihood of surgical complications. A femoro-femoral bypass graft would decrease the patient’s risk of experiencing hemodynamic instability and adverse cardiovascular events during the perioperative process and lower the likelihood of needing another surgery to correct any complications.

## Conclusions

The main goal of an effective physician is to identify disease processes in symptomatic patients and extend medical or surgical care as necessary to provide symptom relief. The sequential identification of dilated cardiomyopathy with triple bypass surgery, seeing evidence of hernioplasty for a large umbilical hernia, a subcutaneous FFBG across the lower abdomen extending between femoral regions, and an abdominal aortic aneurysm with endovascular repair with stent grafts, all providing a challenge to comprehend the series of events that led to pathological findings here and wonder how the patient presented clinically. By disseminating these findings, we strongly motivate students to be curious and vigilant of any such findings during their education and practice and to integrate such findings with relevant clinical presentations so that they can be managed effectively by surgical or medical means to prolong lifespan and provide a better quality of life to such patients in the community.
